# Phosphoenolpyruvate carboxykinase 2 is a promising prognostic biomarker that correlates with peritumoral dendritic cell infiltration in glioblastoma

**DOI:** 10.7150/jca.97034

**Published:** 2025-01-01

**Authors:** Li Yi, Cheng Jiang, Peiquan Guo, Wende Zhu, Xiaobing Jiang

**Affiliations:** Department of Neurosurgery, Union Hospital, Tongji Medical College, Huazhong University of Science and Technology, Wuhan, 430022, China.

**Keywords:** Glioblastoma, PCK2, Immune infiltration, Dendritic cell, Prognostics

## Abstract

**Background:** Despite the growing interest in Phosphoenolpyruvate carboxykinase 2 (PCK2) as a potential biomarker in cancer research, studies on its clinical relevance and biological processes in glioblastoma are still unexplored.

**Methods:** Three main glioma cohorts (TCGA, CGGA, Rembrandt) were extracted to exploit the association between PCK2 expression and clinical relevance through Kaplan-Meier survival analysis, univariate and multivariate cox regression analysis. Immunohistochemistry was used to detect PCK2 expression in glioma samples. GSEA, Pearson correlation and ROC analysis were performed to verify the specificity of PCK2 in mesenchymal GBM. Gene set variation analysis and CIBERSORT were used to explore the correlation of tumor-infiltrating immune cells according to PCK2 expression. Double immunofluorescence was performed to testify the co-expression patterns across PCK2, CD11C and PD-L1 in GBM tissues.

**Results:** PCK2 is increasingly expressed in GBM tissues and could serve as an independent poor prognostic indicator for glioma patients. PCK2 is preferentially expressed in mesenchymal subtype and correlates with immune infiltrates and immunosuppression in glioblastoma. Furthermore, PCK2 exhibits a positive correlation with dendritic cell infiltration and is co-expressed with CD11C and PD-L1 in the peritumoral region of the GBM tissues. Additionally, the enrichment of dendritic cell signature is associated with poor prognosis in glioblastoma patients.

**Conclusion:** Our study highlights the potential therapeutic applicability of PCK2 and PCK2 mediated dendritic cell infiltration as a mechanism for glioblastoma immunosuppression.

## Introduction

Glioblastoma (GBM) is the most common and aggressive primary brain tumor with a dismal outcome^1^. Despite advances in tumor treating field (TTF) based on standard treatment including maximum surgical resection, radiation therapy, and chemotherapy, the median survival time for GBM patients remains disappointingly short, with a median survival of 14-16 months^2^, ^3^. To improve patient outcomes, there is an urgent need for the identification of novel prognostic biomarkers that can aid in risk stratification and the development of targeted therapies^4^, ^5^.

In recent years, Phosphoenolpyruvate carboxykinase 2 (PCK2) has emerged as a potential candidate in cancer research^6^-^8^. PCK2, a key enzyme in gluconeogenesis, plays a critical role in the regulation of cellular energy metabolism, could catalyze mitochondrial oxaloacetate (OAA) to phosphoenolpyruvate (PEP), by its contribution to the PEP pool^9^. PCK2 could give rise to several biosynthetic processes even in the absence of glucose, especially serine and glycerol synthesis. In cancer cells, PCK2 is often overexpressed and contributes to the Warburg effect, a metabolic shift towards aerobic glycolysis even in the presence of oxygen^10^. The hypoxia-inducible factors HIF-1a and EPAS1 could regulate PCK2 expression under glucose limitation and PCK2 is required of glucose-independent cancer cell proliferation and tumor growth *in vivo*. This metabolic rewiring provides cancer cells with a growth advantage, supporting their rapid proliferation upon starvation^11^.

Emerging reports are focus on the link between tumor Warburg metabolism and the immunosuppressive microenvironment^10^, ^12^-^14^. These studies illustrate the similarities and differences among these diverse cell types and how nutrient limitations and molecular cues in the TME promote immune cell dysfunction and regulatory immune cell subsets and provide a niche for tumor maintenance and proliferation^15^-^17^. While PCK2's role in tumor metabolism has been increasingly recognized, its involvement in the modulation of the tumor immune microenvironment remains relatively unexplored. Tumor-infiltrating immune cells play a crucial role in shaping the tumor microenvironment and influencing disease outcome^18^. Dysregulation of the immune system by tumors leads to immunosuppression, allowing cancer cells to evade immune surveillance and clearance^19^-^21^. Recent studies have implicated PCK2 in immune regulation, suggesting a potential link between PCK2 and tumor immunosuppression^22^. Despite the growing interest in PCK2 as a potential biomarker and its links to tumor immunology, research on its relevance in glioblastoma is still poorly defined.

Here, we show that PCK2 is increasingly expressed in GBM tissues and could serve as poor prognostic indicator for glioma patients. PCK2 is preferentially expressed in mesenchymal subtype and correlates with immune infiltrates and immunosuppression in glioblastoma. Furthermore, PCK2 exhibits a positive correlation with dendritic cell infiltration and is co-expressed with CD11C and PD-L1 in the peritumoral region of the GBM tissues. Additionally, the enrichment of dendritic cell signature is associated with poor prognosis in glioblastoma patients. By shedding light on this unexplored area, we hope to stimulate further investigations and pave the way for the development of novel therapeutic strategies targeting PCK2 and the tumor immune microenvironment in GBM.

## Methods and Materials

### The public mRNA expression profile of PCK2 in gliomas

TCGA gliomas mRNA expression profile and clinical data were achieved from cBioPortal (http://www.cbioportal.org/), which includes 539 GBM patients (array platform Affymetrix HT Human Genome U133a) and 702 gliomas (RNAseq data, including 530 low-grade glioma and 172 glioblastoma samples), all of these patients were known with survival data, chemo and radio therapy and other clinical parameters.

CGGA glioma and Rembrandt microarray mRNA expression profile with according to clinical information were achieved from Chinese Glioma Genome Atlas (http://www.cgga.org.cn/). For CGGA glioma dataset includes three sub cohorts which are all LGG and GBM mixed, namely 693 cases of mRNAseq gliomas, 301 cases of microarray gliomas and 325 cases of mRNAseq gliomas. All CGGA dadasets have their paired clinical data (Pathology, grade, age, gender, et.al). For Rembrandt dataset includes 475 cases of glioma patient mRNA microarray data and clinical information like histology, grade, age and OS.

### Exclusion and inclusion criteria

Exclusion and inclusion criteria eligible patients from all the datasets included in this article are in accordance with the following inclusion criteria: 1) pathological confirmation of the diagnosis as gliomas; 2) prior to resection, none of the patients had received any type of therapy, including chemotherapy, radiation, or immunotherapy; Exclusion criteria included the following: 1) Other types of tumors. 2) uncomplete clinicopathological data. The detailed clinic parameters of enrolled patients for clinical feature analysis were presented in Supplementary Table 1.

### Clinical glioma samples

Fresh glioma tissues and adjacent non-tumorous tissues were acquired from the from the patients operated at the Department of Neurosurgery, Wuhan Union Hospital (Wuhan, China). Formalin-fixed, paraffin-embedded glioma tissues and correlative clinicopathological information were also collected from Wuhan Union Hospital.

### IHC and IF staining

Immunohistochemistry (IHC) staining of paraffin-embedded glioma tissues with antibody against PCK2 (1:200, 14892-1-AP Proteintech) was performed following the standard procedures as previously described^23^. The staining intensity was scored as 0 (negative), 1 (weak), 2 (moderate), or 3 (strong). The percentage of positive cells was scored as 1 (1-25%), 2 (26-50%), 3 (51-75%), or 4 (76-100%). A final immunoreactivity score (IRS) of 0-12 was obtained by multiplying the intensity and percentage scores.

Tyramide Signal Amplification (TSA) method was used for multiplex immunofluorescence (mIF) staining of paraded GBM tissues with antibody against PD-L1 (1:100, 66248-1-Ig, Proteintech), CD11c (1:100, 17342-1-AP, Proteintech) and PCK2 was performed according to manufacturer´s instructions. Cy3 Goat Anti-Rabbit IgG H&L secondary antibody (1:200, abcam) and Alexa-Fluor 488 Goat Anti-Rabbit IgG H&L secondary antibody (1:500, abcam, USA), Goat Anti-Rabbit IgG H&L (HRP) (1:2000, abcam) were used for fluorescent double-staining. ImageJ was used for the quantification and visualization of colocalized signals.

### Clinical feature and Cox regression analysis

Glioma patients from CGGA (n=693) and TCGA (n=702) datasets were accessed to perform cox regression analysis. Patients with incomplete data are removed from the analysis. The Cox proportional hazards regression model was employed to investigate the prognostic factors associated with the time-to-event outcome in this study. To achieve this, both univariate and multivariate Cox analyses were performed. Initially, univariate Cox analysis was conducted on each potential predictor individually. This allowed for the identification of significant associations between individual predictors and the event of interest, providing valuable insights into their unadjusted impact on the survival outcome.

Subsequently, the statistically significant predictors from the univariate analysis were selected for inclusion in the multivariate Cox analysis. This step aimed to explore the independent contributions of these factors while adjusting for the influence of other covariates. The multivariate Cox analysis allowed us to identify the predictors that remained significantly associated with the survival outcome after accounting for potential confounding variables.

Hazard ratios (HRs) along with their corresponding 95% confidence intervals (CIs) were calculated to quantify the strength and direction of the associations in both univariate and multivariate models. Statistical significance was determined using a significance level of p < 0.05. All analyses were performed using SPSS to appropriately handle censored observations and time-to-event data.

### Immune cell infiltration analysis

TIMER web server is a comprehensive resource for systematical analysis of immune infiltrates across diverse cancer types. The abundances of six immune infiltrates (B cells, CD4+ T cells, CD8+ T cells, Neutrophils, Macrophages, and Dendritic cells) are estimated by TIMER algorithm.

Single-sample Gene Set Enrichment Analysis (ssGSEA) is a computational method used to evaluate the enrichment of immune cell infiltration in individual samples.

### Gene set enrichment analysis

GSEA software (version 4.3.2) was applied to perform GSEA to investigate meaningful association between PCK2 expression and GBM subtype signatures. Pathways with nominal p-value < 0.05 and FDR < 0.25 were considered significantly enriched.

To estimate the population specific immune infiltration, we used single sample gene set enrichment analysis (ssGSEA) that define an enrichment score to represent the degree of absolute enrichment of a gene set in each sample within a given dataset^16^. Normalized enrichment scores could be calculated for each immune category. The ssGSEA analysis were performed in R package GSVA.

## Results

### PCK2 is highly expressed in GBM and correlates with shorter OS

**Figure 1:**
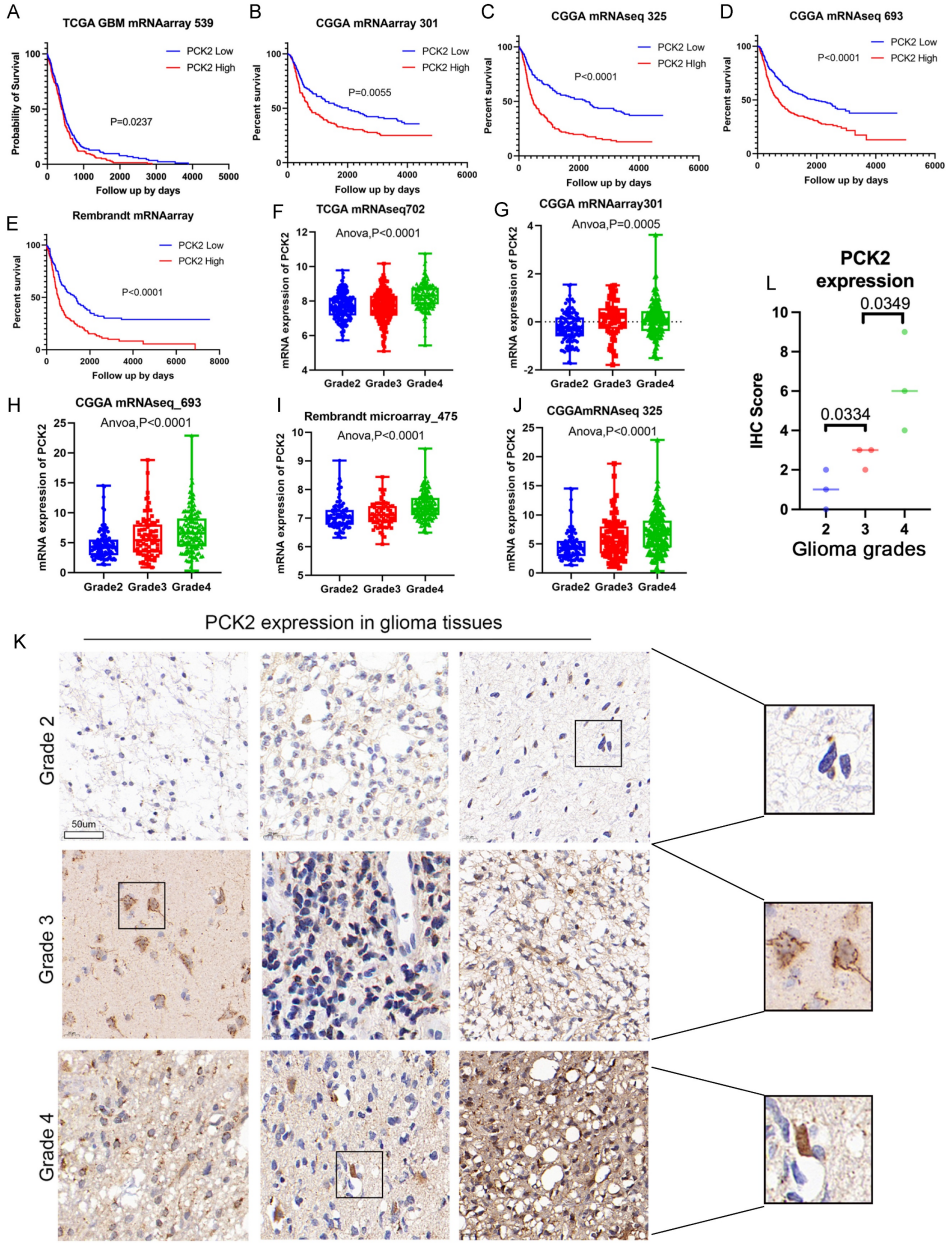
** The OS relation and expression of PCK2 in glioma patients. A-E.** The correlation between PCK2 mRNA expression and glioma patient overall survival time (days) across five TCGA, CGGA and Rembrandt datasets (median value as threshold). **F-J.** The PCK2 mRNA expression in different grades of glioma patients based on five TCGA, CGGA and Rembrandt datasets (One-way ANOVA analysis). **K-L.** Immunohistochemical staining of PCK2 in glioma tissue specimens (Grade2-4) with their IHC score indicated.

The analysis of multiple glioma mRNA expression cohorts from three major glioma related databases, TCGA, CGGA, and Rambrant, revealed that glioma patients with high PCK2 expression (median value threshold) have a poorer prognosis and shorter overall survival compared to those with low PCK2 expression (Log-rank test, P<0.05) (Fig. 1A-E). Additionally, the examination of different glioma grades demonstrated that PCK2 expression generally increases with tumor grade, suggesting a positive correlation between PCK2 levels and glioma progression (One-way ANOVA, P<0.05) (Fig. 1F-J). Furthermore, these findings were validated in nine human glioma tissues at the protein level through immunohistochemistry staining, as compared to lower grade gliomas (grade 2 and 3), PCK2 is significantly highly expressed in GBM patients (Fig. 1K-L, Fig. S1). These results highlight the high expression of PCK2 in glioma patients related to tumor progression and poor prognosis.

### PCK2 serves as an independent prognostic biomarker in glioma

The mRNA expression level of PCK2 was investigated in glioma samples retrieved from the CGGA and TCGA databases. The gene expression data were normalized, and chi-square test and t test were used to compare the expression levels between PCK2 high expressed patient and PCK2 low expressed patient based on prognosis or other factors. The results revealed significant differences in IDH1 mutation and 1P19q codeletion status between PCK2 low and PCK2 high group (p-value < 0.05) in both two datasets, indicating a potential association of high PCK2 expression with IDH1 wildtype or 1P19Q non codeletion (Table 1 and 2), which are both unfavorable clinical characteristics for glioma patients.

To further assess the prognostic significance of PCK2, univariate Cox regression analysis was performed using SPSS software (Table 3 and 4). The analysis incorporated age, gender, IDH1, 1P19q, MGMT, ATRX, as well as PCK2 expression. The results indicated IDH1 mutate, 1P19 codeletion, ATRX and MGMT as robust favorable clinical prognostic indicators, while PCK2 along with age (PCK2, TCGA: P<0.0001, CGGA: P<0.0001) serve as indicators for poor survival time in glioma patients. To ascertain whether the prognostic significance of PCK2 is independent of other clinical variables, multivariate Cox regression analysis was performed. The covariates included the significant variables previously verified in univariate analysis. The results demonstrated that PCK2 expression remained significantly associated with shorter OS after adjusting for the other covariates (TCGA: P=0.022, CGGA: P=0.001).

### Preferential Expression of PCK2 on mesenchymal GBM

**Figure 2:**
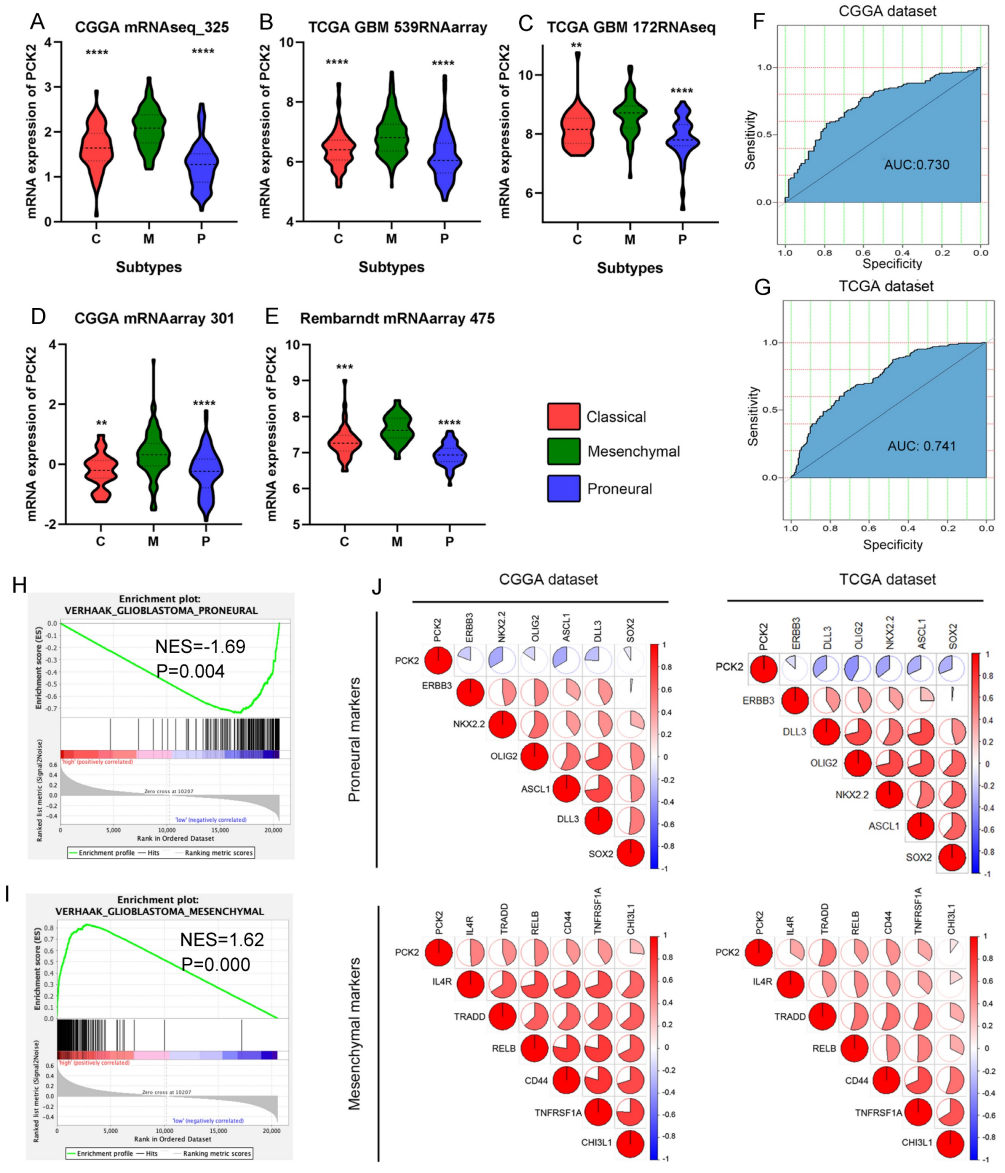
** PCK2 is preferentially expressed in mesenchymal subtype of GBM. A-E.** The mRNA expression of PCK2 in classical, mesenchymal and proneural GBMs based on five datasets from CGGA, TCGA and Rembrandt cohorts. **F-G.** Receiver operating characteristic (ROC) curve analysis showed the sensitivity and specificity of PCK2 to predict mesenchymal subtype in TCGA and CGGA database. **H-I.** GSEA analysis showed the association between PCK2 and proneural or mesenchymal signature in GBM. **J.** The Pearson correlation analysis showed the association between PCK2 and proneural or mesenchymal markers across CGGA and TCGA datasets.

Depending on molecular characteristics, GBM is classified into three subtypes, including the proneural, classical, and mesenchymal subtypes. Different subtypes represent distinct tumor genetic and phenotypic natures, and prognoses, with the proneural subtype having the best prognosis and the mesenchymal subtype having the worst prognosis. Analysis of TCGA, CGGA, and Rembrandt databases reveals that PCK2 expression is significantly higher in mesenchymal GBM compared to the proneural and classical subtypes (Fig. 2A-E). ROC curve analysis shows that PCK2 expression can effectively distinguish the mesenchymal subtype from other subtypes, higher PCK2 expression indicates a greater likelihood of the specimen being classified as the mesenchymal subtype (CGGA: AUC:0.730, TCGA: AUC:0.741) (Fig. 2F-G).

GSEA analysis demonstrates a positive correlation between high PCK2 expression and the gene set of the mesenchymal subtype signature in glioma patients, while a negative correlation with the proneural signature (Fig. 2H-I). Pearson correlation analysis also indicates a negative correlation between PCK2 and proneural subtype markers such as ERBB3, NKX2-2, OLIG2, ASCL1, DLL3, SOX2, and a positive correlation with mesenchymal subtype markers such as IL4R, TRADD, RELB, and CD44. Taking together, PCK2 is strongly associated with mesenchymal glioblastoma and can serve as a potential biomarker for this subtype (Fig. 2J).

### Correlation between PCK2 and immune infiltration in GBM

**Figure 3:**
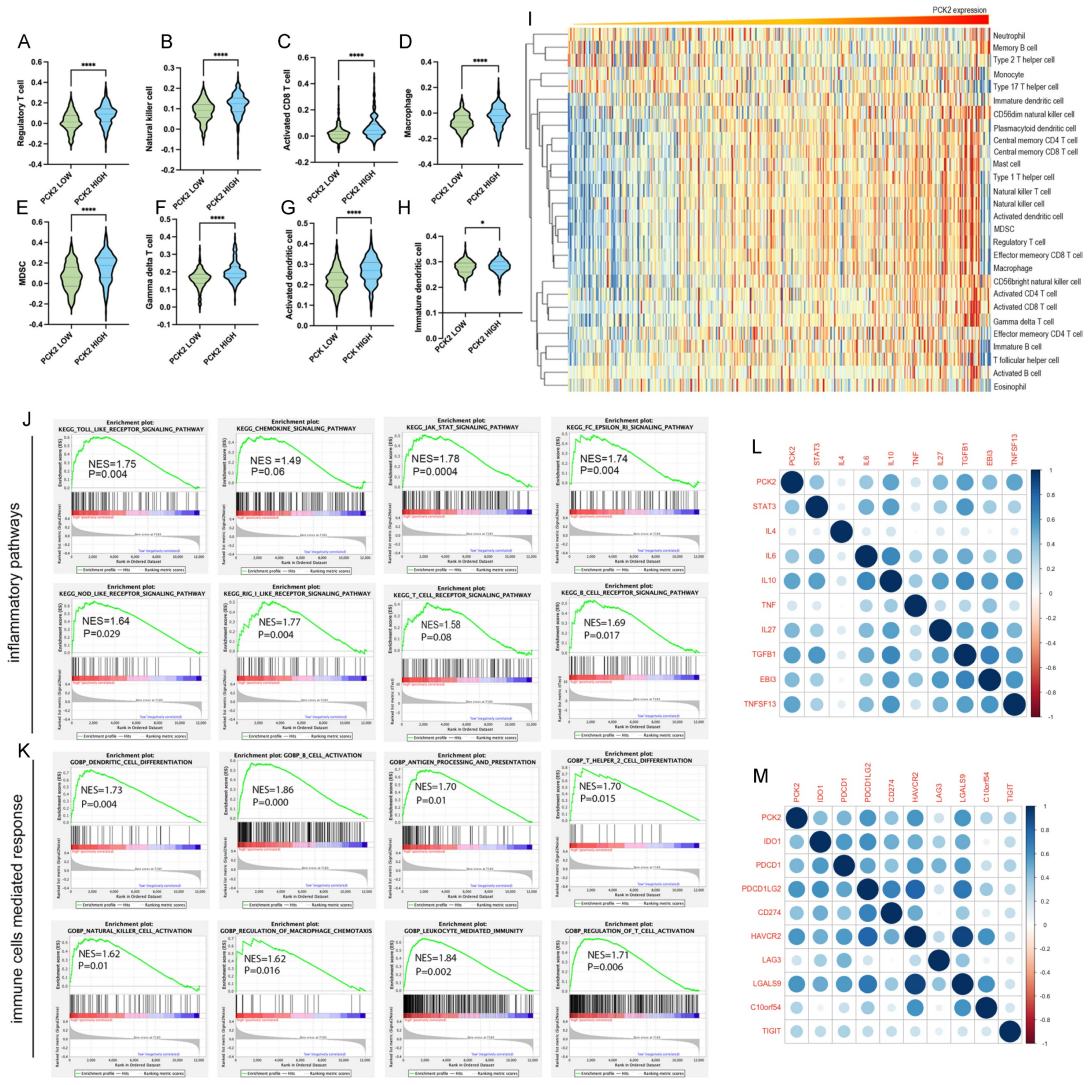
** PCK2 correlates with immune infiltration in GBM. A-H.** ssGSEA analysis displayed the association between PCK2 expression level and different immune cell types. (*:P<0.05, ****:P<0.0001) **I.** The heatmap for the fraction of the different immune cell types indicated by PCK2 expression. **J.** GSEA analysis shows the association between inflammatory pathways and high PCK2 expression of GBM patients. **K.** GSEA analysis shows the association between immune cells mediated responses and high PCK2 expression of GBM patients. **L-M** Pearson correlation analysis shows the association of PCK2 with inflammatory factors and immune checkpoints in GBM.

Mesenchymal glioblastoma is distinguished by its markable invasiveness, extensive necrosis, and abundant immune infiltration. The ssGSEA method was utilized to analyze the correlation between PCK2 expression and the infiltration of different immune cells. The results revealed that high expression of PCK2 in glioblastoma indicates a higher proportion of infiltrating T reg cells, natural killer cells, myeloid-derived suppressor cells (MDSCs), CD8+ T cells, dendritic cells, and macrophages (Fig. 3A-H). Furthermore, the heatmap analysis demonstrated that the majority of immune cells exhibited a positive correlation with PCK2 expression levels (Fig. 3I). Simultaneously, we utilized GSEA analysis to explore the enrichment of inflammatory response pathways and immune cell responses in patients with high and low PCK2 expression. The findings demonstrated that glioblastoma patients with high PCK2 expression showed substantial enrichment in various immune response pathways, including Toll-like, JAK, NOD-like, RIG, and Fc epsilon RI signaling (Fig. 3J). Moreover, they exhibited heightened enrichment in cell-mediated immune responses, such as B cell activation, dendritic cell differentiation, macrophage activation, and NK cell activation (Fig. 3K). Furthermore, Pearson correlation analysis revealed that PCK2 is positively correlated with immune inhibitory factors (STAT3, IL4, IL10, TNF, TGFB1) (Fig. 3L) and immune checkpoint molecules (PDCD1, PD-L1, IDO1, PD-L2) (Fig. 3M).

### PCK2 expression relates to DCs infiltration and immunosuppression in GBM

**Figure 4:**
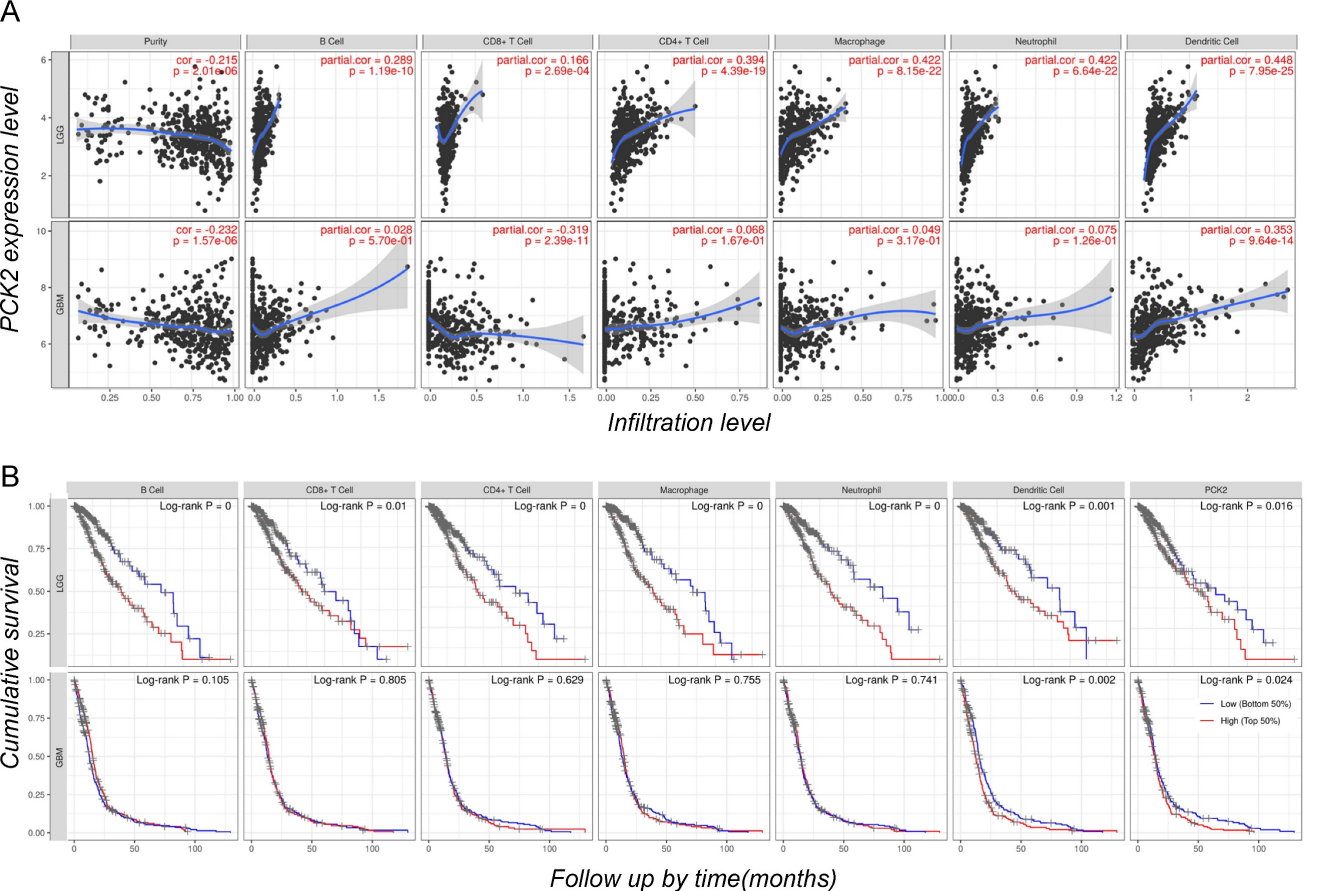
** The immune infiltrates and outcome of PCK2 in LGG and GBM. A**. Correlation of PCK2 expression with tumor purity and various immune cells. **B.** Kaplan-Meier plots for immune infiltrates and PCK2 to visualize the survival differences (median value as threshold).

To further investigate the correlation between PCK2 and different immune cells, as well as the association between immune cell infiltration and glioblastoma prognosis, we conducted an analysis using the CIBERSORT based TIMER web tool. The results revealed that among several major immune cell types including B cells, CD4 and CD8 T cells, macrophages, neutrophils, and dendritic cells. The expression of PCK2 exhibits a strong positive correlation with dendritic cells, while demonstrating a negative correlation with CD8 T cells and tumor purity (Fig. 4A). Meanwhile, cumulative survival analysis demonstrated that strong immune infiltration with poor survival time in LGG patients, while in GBM patients only dendritic cells showed a consistent trend, which is similar to PCK2 (Fig. 4B).

**Figure 5:**
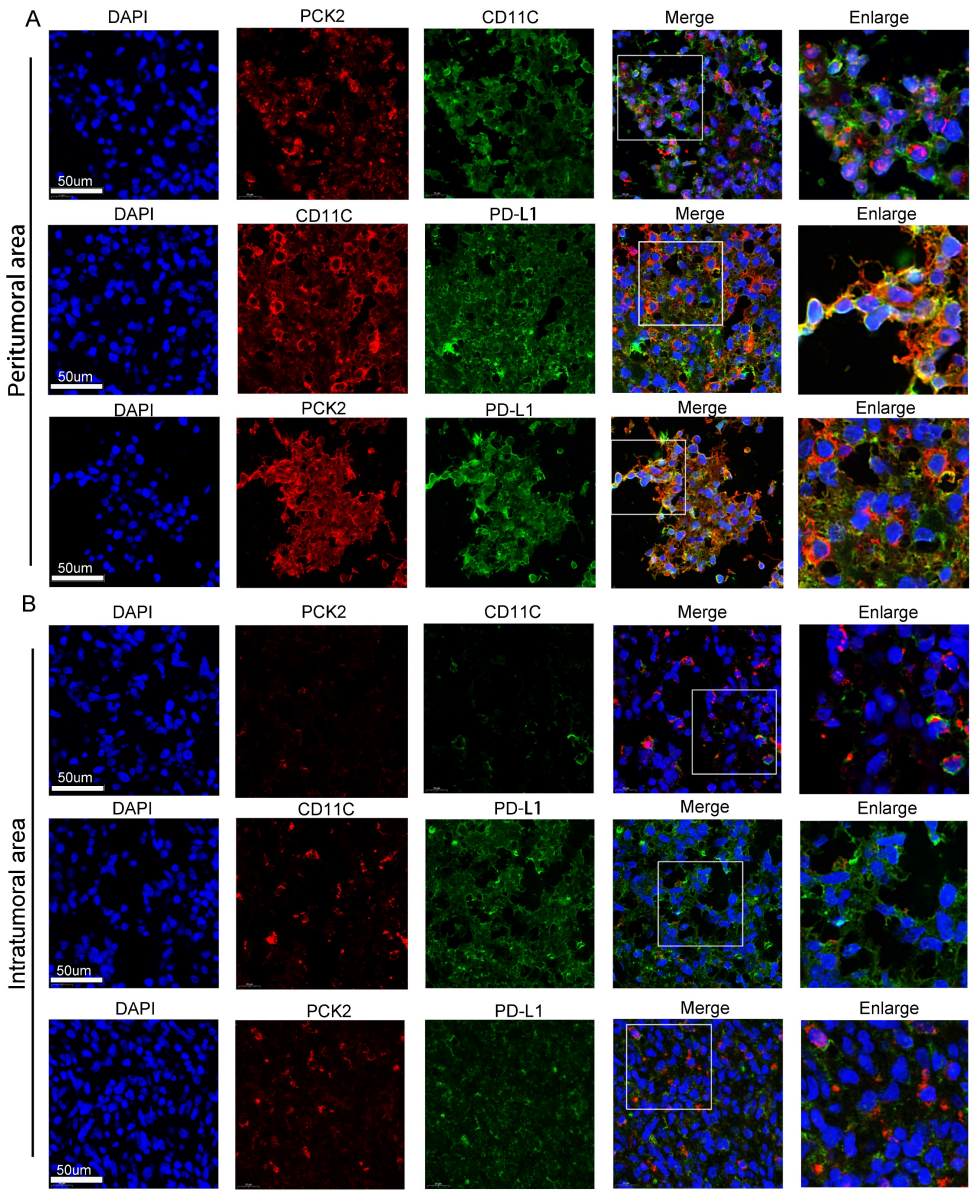
** Immunofluorescent staining of PCK2, CD11C and PD-L1 in GBM samples. A.** The coexpression of PCK2, CD11C and PD-L1 in peritumor area of GBM tissues. **B**. The coexpression of PCK2, CD11C and PD-L1 in intratumor area of GBM tissues.

To gain deeper exploration into the association between PCK2 and dendritic cells, as well as its impact on immune suppression, we pursued samples from glioblastoma multiforme tumors. We meticulously examined the PCK2, CD11C, and PD-L1 expressions within both the central tumor core and the tumor-infiltrated peripheral regions through multiplex staining. The results showed that compared to the tumor center region (Fig. 5B), higher expressions of PCK2, CD11C, and PDL1 were detected in the tumor-infiltrated area (Fig. 5A). Among these markers, PCK2 owns a particularly high correlation with CD11C expression in peritumor area (R=0.8026) versus intratumoral area (R=0.3149) (Fig. S2).

### Tumor associated DCs predicts poor prognosis in GBM

**Figure 6:**
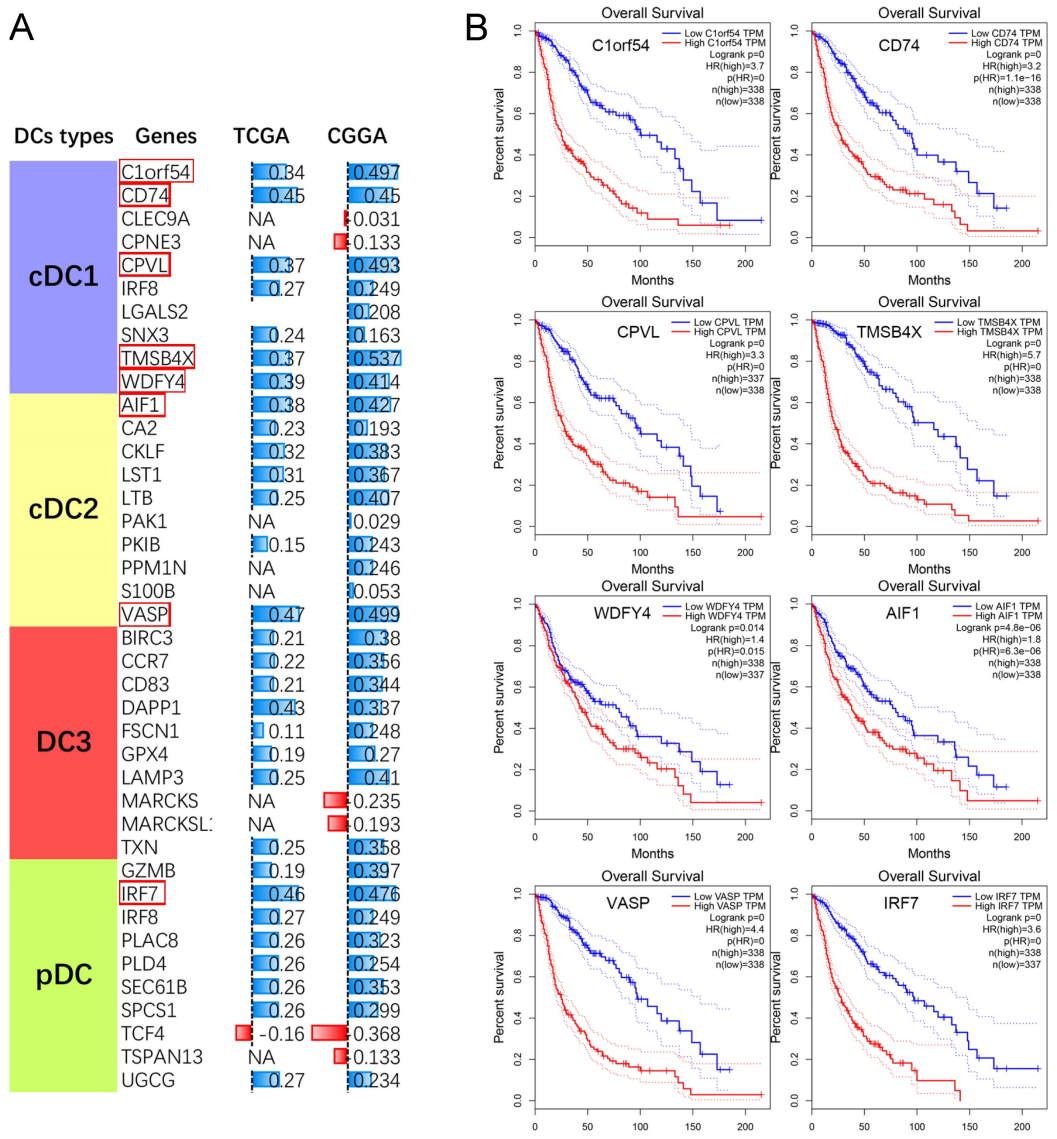
** The association between PCK2 and DCs in GBM. A.** The Pearson correlation analyses reveal the correlation between PCK2 and DCs subtype markers. (NA: No significant differences) **B.** The Kaplan-Meier survival plot of PCK2 most related DCs markers in glioma patients based on TCGA dataset (median value as threshold).

Meanwhile, we also performed a correlation analysis between PCK2 and genes associated with different subtypes of tumor-infiltrating dendritic cells. The results revealed a strong positive correlation between PCK2 and characteristic genes of different subtypes of dendritic cells (Fig. 6A). Subsequently, we performed survival analysis on the top 8 genes most correlated with PCK2 based on TCGA dataset. The results indicated that the expression of these genes is indicative of poor prognosis in glioma patients (Fig. 6B).

## Discussion

The interplay between the tumor and the immune infiltration within the tumor microenvironment significantly impacts disease, therapy response, and patient outcomes^24^-^26^. Dendritic cells (DCs), while constituting a minority within the tumor microenvironment, are gaining recognition as a vital element in the fight against tumors, as they are central for the initiation of antigen-specific T cell immunity and tolerance^27^, ^28^.

Our study sheds light on the multifaceted role of PCK2 in glioblastoma, unraveling its significance as a poor prognostic factor and its strong associations with immune cell infiltration, particularly tumor associated dendritic cells. This emphasizes its potential as an independent unfavorable prognostic indicator, suggesting its utility as a diagnostic marker and a candidate for therapeutic targeting. Intriguingly, PCK2 is preferentially expressed in the mesenchymal GBM, accompanied by a positive correlation with immune cell infiltration, underscores its potential involvement in shaping the immune landscape within the tumor microenvironment.

Under the pressure of antitumor immunity, cancer cells could promote immune tolerance by presenting tumor- associated antigens (TAAs) on MHC molecules and providing co-stimulation and soluble factors. Our studies demonstrated that PCK2 is strongly associated with dendritic cells (DCs) markers underling its potential impact on immune modulation. The co-expression of PCK2 with dendritic cell markers, such as CD11C, along with the co-expression of the immune checkpoint PD-L1, implies a potential association between PCK2 and tumor associated DCs mediated immunosuppression. It suggests that PCK2 might contribute to dendritic cell infiltration and phenotypic change, possibly inhibiting the initiation and progression of immune responses against the tumor.

The implications of our findings extend to the broader context of immunotherapy strategies in GBM treatment. The correlation between PCK2 and immune checkpoints raises questions about potential immune evasion mechanisms driven by PCK2, which could hinder the efficacy of immune checkpoint blockade therapies. Moreover, the potential involvement of PCK2 in dendritic cell infiltration suggests a novel avenue for therapeutic interventions. Strategies targeting PCK2 could potentially enhance dendritic cell-based immunotherapies, thus restoring and augmenting anti-tumor immune responses.

## Conclusions

In conclusion, our study sheds light on the multifaceted role of PCK2 in glioblastoma, encompassing both its prognostic significance and its potential influence on immune modulation. These findings warrant further investigation into the mechanistic underpinnings of PCK2's effects on dendritic cell function and immune responses, potentially paving the way for innovative therapeutic approaches in the realm of glioblastoma treatment and immunotherapy.

## Supplementary Material

Supplementary figures.

## Figures and Tables

**Table 1:** Analysis of PCK2 expression in CGGA gliomas and associated clinicopathological factors

Characteristics	PCK2 expression		P value
	Low (n=207)	High (n=206)	
Gender			0.0152
Male	101 (49%)	125 (60.7%)	
Female	106 (51%)	81 (39.3%)	
Age, years			0.0535
<60	191(92.3%)	178(86.4%)	
≥60	16(7.7%)	28(13.6%)	
OS time average	1400.6 days	1048.6 days	0.0005
OS event			0.0003
Alive	92 (44.4%)	56 (27.2%)	
Dead	115 (55.6%)	150 (72.8%)	
Histology			
AA	36(17.4%)	22(10.7%)	
O	18(8.7%)	6(2.9%)	
A	24(11.6%)	23(11.2%)	
AO	21(10.1%)	8(3.9%)	
GBM	37(17.9%)	53(25.7%)	
rA	7 (3.4%)	8(3.9%)	
rO	2 (1.0%)	8(3.9%)	
rAA	27(9.7%)	25(12.1%)	
rAO	15(7.2%)	7(3.4%)	
rGBM	20(9.7%)	46(22.3%)	
PRS type			0.0187
Primary	136(65.7%)	112(54.4%)	
Recurrent	71(34.3%)	94(45.6%)	
WHO Grade			
2	51(24.6%)	45(21.8%)	
3	99(47.8%)	62(30.1%)	
4	57(27.5%)	99(48.1%)	
IDH1 Mutation			<0.0001
Mutated	141(68.1%)	87(42.2%)	
WT	66(31.9%)	119(57.8%)	
1p19q status			<0.0001
Codel	62(30.0%)	25(12.1%)	
Non-Codel	145(70.0%)	181(87.9%)	
MGMTp_methylation			0.5893
methylated	125(60.4%)	119(57.8%)	
un-methylated	82(39.6%)	87(42.2%)	

**Table 2:** Analysis of PCK2 expression in TCGA gliomas and associated clinicopathological factors

Characteristics	PCK2 expression		P Value
	Low (n=102)	High (n=101)	
Gender			0.5238
Male	52(51.0%)	56(55.4%)	
Female	50(49.0%)	45(44.6%)	
Age, years			0.3631
<60	84(82.4%)	78(77.2%)	
≥60	18(17.6%)	23(22.8%)	
OS average, month	28.9	23.7	0.2312
OS event			0.1133
Alive	77(75.5%)	66(65.3%)	
Dead	25(24.5%)	35(34.7%)	
Histology			
A	5(4.9%)	13(12.9%)	
O	27(26.5%)	16(15.8%)	
OA	9(8.8%)	10(10.0%)	
AA	22(21.6%)	26(25.7%)	
AO	22(21.6%)	14(13.9%)	
AOA	11(10.8%)	9(9.0%)	
GBM	6(5.9%)	13(12.9%)	
WHO grade			
2	41(40.2%)	39(38.6%)	
3	55(53.9%)	49(48.5%)	
4	6(5.9%)	13(12.9%)	
IDH1 mutation			0.0004
Mutated	86(84.3%)	63(62.4%)	
WT	16(15.7%)	38(37.6%)	
1q19q			0.0006
Codel	38(37.3%)	16(15.8%)	
Non-codel	64(62.7%)	85(84.2%)	
MGMT			0.0404
Methylated	85(83.3%)	72(71.3%)	
Un-methylated	17(16.7%)	29(28.7%)	
TERT Promoter			0.3620
Mutated	47(46.1%)	53(52.5%)	
WT	55(53.9%)	48(47.5%)	
ATRX			0.6250
Mutation	37(36.3%)	40(39.6%)	
WT	65(63.7%)	61(60.4%)	

**Table 3:**
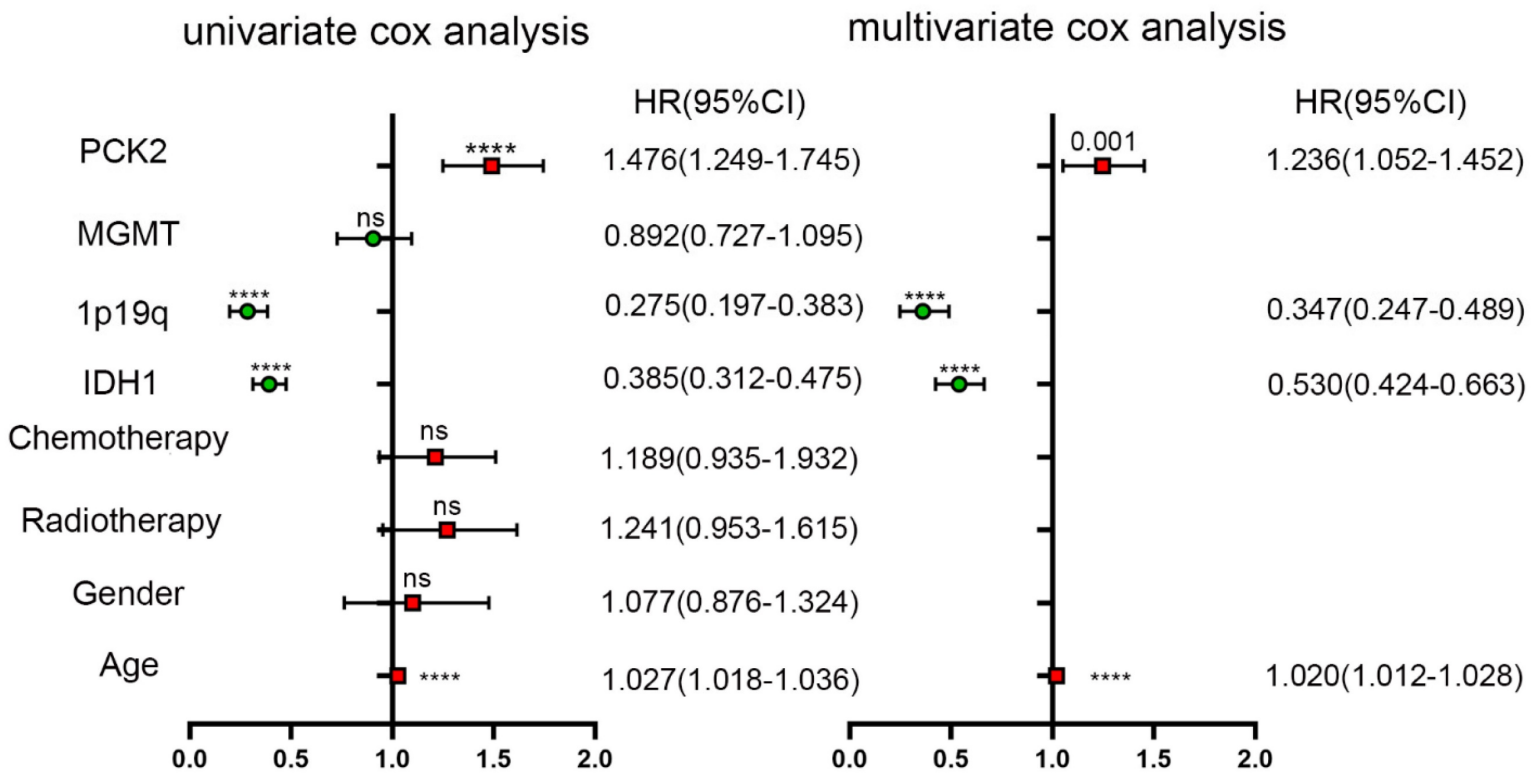
Univariate and multivariate analyses of PCK2 and other variables in CGGA dataset

**Table 4:**
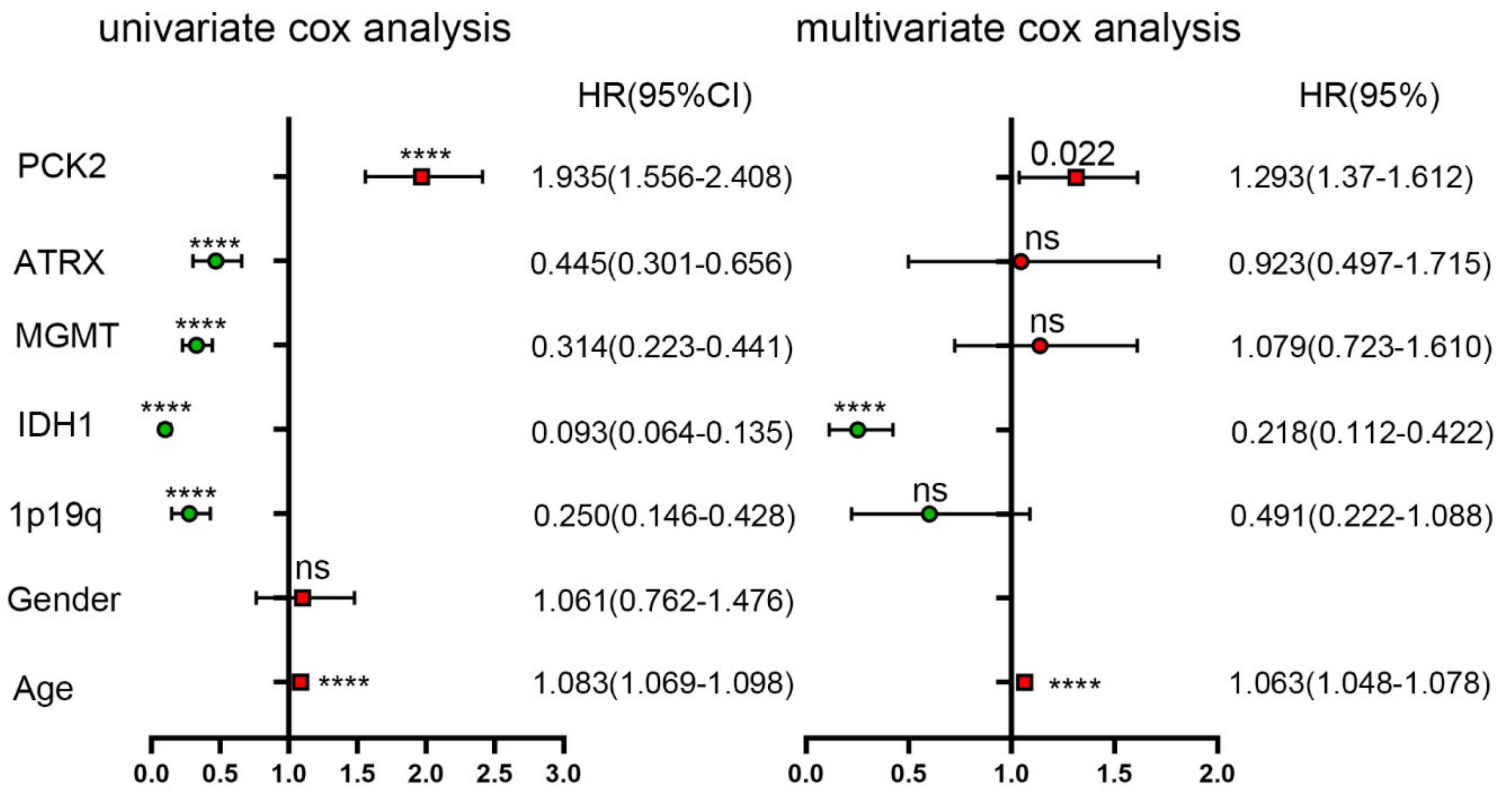
Univariate and multivariate analyses of PCK2 and other variables in TCGA dataset

## Abbreviations

GBM: Glioblastoma
LGG: Lower grade glioma
DCs: Dendritic cells
PCK2: Phosphoenolpyruvate carboxykinase 2
TAAs: Tumor associated antigens
TME: tumor microenvironment
IHC: Immunohistochemistry
ssGSEA: single sample gene set enrichment analysis
TTF: Tumor treating field
TIDC: Tumor-infiltrating dendritic cells
PEP: phosphoenolpyruvate
