# Supplementary material for: Phosphoenolpyruvate carboxykinase 2 is a promising prognostic biomarker that correlates with peritumoral dendritic cell infiltration in glioblastoma
Source: J Cancer. 2025 Jan 1;16(2):590–602. doi: 10.7150/jca.97034 (PMC11685687; doi:10.7150/jca.97034)
Supplement: Supplementary file 1 — Supplementary figures. [file jcav16p0590s1.pdf]

Supplementary Figure1

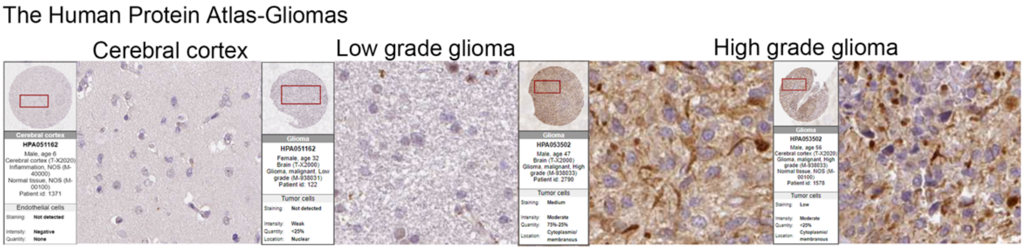

Detection of PCK2 expression in tissues of glioma patients and cerebral cortex from HPA database.

Supplementary Figure2

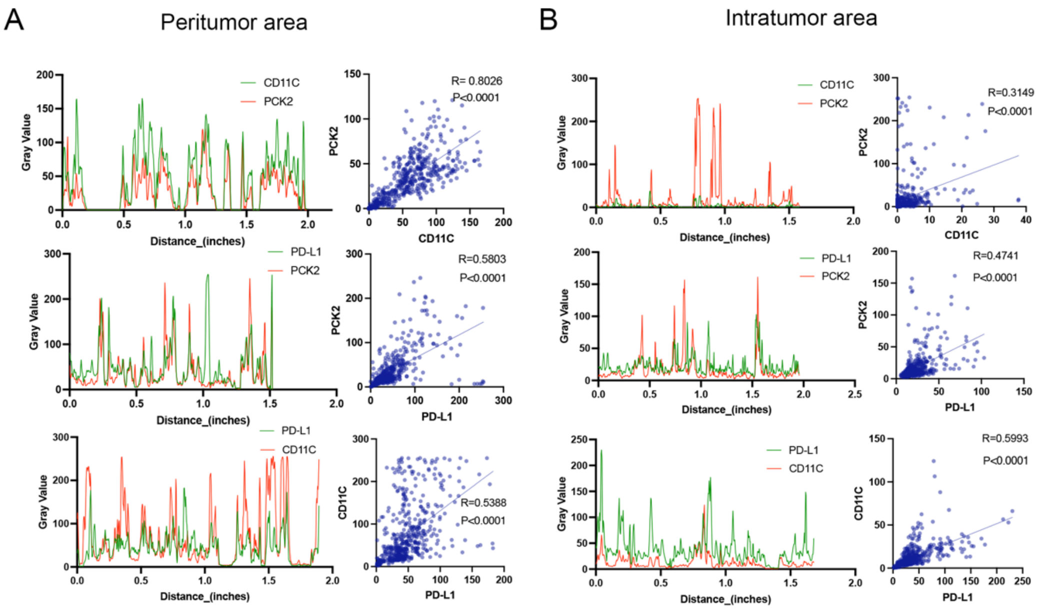

A-B. Quantification of PCK2, CD11C and PD-L1 coexpression in GBM peritumor area and intratumor area with correlation coefficient (R) indicated.
